# The transcription factor MITF is a critical regulator of GPNMB expression in dendritic cells

**DOI:** 10.1186/s12964-015-0099-5

**Published:** 2015-03-24

**Authors:** Michael Gutknecht, Julian Geiger, Simone Joas, Daniela Dörfel, Helmut R Salih, Martin R Müller, Frank Grünebach, Susanne M Rittig

**Affiliations:** Department of Internal Medicine II, Oncology, Hematology, Immunology, Rheumatology and Pulmology, University of Tübingen, Otfried-Müller-Str. 10, 72076 Tübingen, Germany

**Keywords:** Dendritic cells, Coinhibitory receptor, Glycoprotein NMB, Tyrosine kinase inhibitors, PI3K/Akt signaling pathway, Microphthalmia-associated transcription factor

## Abstract

**Background:**

Dendritic cells (DC) are the most potent antigen-presenting cells (APC) with the unique ability to activate naïve T cells and to initiate and maintain primary immune responses. Immunosuppressive and anti-inflammatory stimuli on DC such as the cytokine IL-10 suppress the activity of the transcription factor NF-κB what results in downregulation of costimulatory molecules, MHC and cytokine production. Glycoprotein NMB (GPNMB) is a transmembrane protein, which acts as a coinhibitory molecule strongly inhibiting T cell responses if present on APC. Interestingly, its expression on human monocyte-derived dendritic cells (moDC) is dramatically upregulated upon treatment with IL-10 but also by the BCR-ABL tyrosine kinase inhibitors (TKI) imatinib, nilotinib or dasatinib used for the treatment of chronic myeloid leukemia (CML). However, the molecular mechanisms responsible for GPNMB overexpression are yet unknown.

**Results:**

The immunosuppressive cytokine IL-10 and the BCR-ABL TKI imatinib or nilotinib, that were examined here, concordantly inhibit the PI3K/Akt signaling pathway, thereby activating the downstream serine/threonine protein kinase GSK3ß, and subsequently the microphthalmia-associated transcription factor (MITF) that is phosphorylated and translocated into the nucleus. Treatment of moDC with a small molecule inhibitor of MITF activity reduced the expression of GPNMB at the level of mRNA and protein, indicating that GPNMB expression is in fact facilitated by MITF activation. In line with these findings, PI3K/Akt inhibition was found to result in GPNMB overexpression accompanied by reduced stimulatory capacity of moDC in mixed lymphocyte reactions (MLR) with allogeneic T cells that could be restored by addition of the GPNMB T cell ligand syndecan-4 (SD-4).

**Conclusions:**

In summary, imatinib, nilotinib or IL-10 congruently inhibit the PI3K/Akt signaling pathway thereby activating MITF in moDC, resulting in a tolerogenic phenotype. These findings extend current knowledge on the molecular mechanisms balancing activating and inhibitory signals in human DC and may facilitate the targeted manipulation of T cell responses in the context of DC-based immunotherapeutic interventions.

**Electronic supplementary material:**

The online version of this article (doi:10.1186/s12964-015-0099-5) contains supplementary material, which is available to authorized users.

## Background

Pathways involved in negative T cell regulation are of great interest, as on the one hand they can fatally attenuate T cell responses against cancer cells and on the other hand do offer an opportunity to develop tolerance-inducing strategies in Graft-versus-host disease (GvHD) and autoimmune diseases [1,2].

DC are the most powerful APC and play a key role in balancing T cell responses, depending on their expression of costimulatory and/or coinhibitory molecules [3]. After stimulation by TLR ligands, TNF, IFN-γ or T cell signals, DC undergo a complex maturation process, express costimulatory molecules and migrate into lymph nodes where they prime naive T cells. In contrast, in the absence of activating signals and/or in the presence of immunosuppressive and anti-inflammatory factors like IL-10, TGF-β, prostaglandin D2 (PGD2) or corticosteroids, DC achieve a tolerogenic phenotype mediated by the expression of molecules that suppress T cell activation and induce T cell anergy [3,4]. Due to their unique ability to induce specific T cell responses DC are employed in immunotherapeutic strategies against cancer aiming at the induction of long term clinical responses [5-7].

At the same time, targeted therapies with TKI have significantly improved treatment of cancer with imatinib being the first to be established in the treatment of chronic myeloid leukemia (CML). It efficiently blocks the pathologically activated c-ABL tyrosine kinase activity of the BCR-ABL fusion oncogene [8-10]. Nilotinib and dasatinib, second-generation TKI initially developed for the treatment of patients who are resistant or intolerant to imatinib, are now used as first-line therapy [11-13]. Besides c-ABL, these TKI significantly inhibit c-Kit and PDGFR tyrosine kinase activity and imatinib therefore is being used against other malignancies including gastrointestinal stromal tumors. However, little is known about their effects on immune cells.

Recently, the type I transmembrane receptor GPNMB (Glycoprotein NMB, DC-associated transmembrane protein (DC-HIL), osteoactivin), expressed on APC, was shown to strongly inhibit responses of CD4^+^ and CD8^+^ T cells by binding to its ligand syndecan-4 (SD-4) [14-18]. We previously demonstrated that primary human moDC moderately express GPNMB and dramatically upregulate its expression if generated in the presence of the cytokine IL-10, a main suppressor of cellular immunity, but notably also when exposed to imatinib, nilotinib or dasatinib [19,20].

Here we aimed to elucidate the molecular switch of cellular signaling upon inhibition of moDC. Our *in vitro* study revealed concordant inhibition of PI3K/Akt signaling by IL-10 or the BCR-ABL TKI imatinib and nilotinib that resulted in dephosphorylation and activation of glycogen synthase kinase-3-ß (GSK3ß) and subsequent phosphorylation and translocation of the transcription factor MITF [21]. Moreover, treatment of moDC with the small molecule inhibitor of the MITF molecular pathway ML329 [22] reduced the expression of GPNMB at the level of mRNA and protein, indicating that GPNMB expression is in fact facilitated by MITF activation.

The basic helix-loop-helix leucine zipper transcription factor MITF, which was initially described as a key regulator for melanocyte differentiation, comprises at least eight isoforms differentially expressed within various cell types [21,23]. However, its expression pattern and functional role in hematopoietic and blood cells was so far unknown.

Finally, PI3K/Akt inhibition was found to result in GPNMB overexpression accompanied by reduced stimulatory capacity of moDC in mixed lymphocyte reactions (MLR) with allogeneic T cells that could be restored by addition of the T cell ligand SD-4, demonstrating the functional relevance of the elucidated signaling mechanism.

Taken together, our data indicate that the therapeutically used BCR-ABL TKI imatinib and nilotinib exert immunosuppressive effects in primary moDC by interfering with pathways involved in IL-10 receptor signaling and activation of MITF. These findings extend the current knowledge about the molecular mechanisms balancing between activating and inhibitory signals in DC and, thus, could help to avoid impaired immune responses due to TKI treatment. In addition, manipulation of the relevant signaling cascades and/or GPNMB expression or function may constitute a promising strategy in combinatory approaches using BCR-ABL TKI and DC-based immunotherapy and may also allow for manipulation of T cell responses in GvHD.

## Results

### PI3K/Akt-Inhibition upregulates GPNMB expression in moDC

Besides BCR-ABL, imatinib, nilotinib and dasatinib inhibit a variety of other kinases including c-Kit [24]. The main downstream signaling cascades are the Ras/Erk- and the PI3K/Akt pathway. Evidence that IL-10 receptor signaling could be affected by these clinically used TKI is deduced from the observation in mouse DC that IL-10 blocks Akt phosphorylation, and inhibitors of PI3K effectively suppress the activation of Akt and subsequent IκB kinase (IKK) and nuclear factor-κB (NF-κB) [25].

In our first experiments, the relevance of these pathways in (up-) regulation of immune repressive GPNMB in human DC was examined. Therefore, we generated immature moDC *in vitro* from CD14^+^ monocytes of healthy donors, incubated with the PI3K inhibitor LY294002, Akt inhibitor MK2206, Erk inhibitor FR180204 or imatinib or nilotinib as a control. GPNMB expression was determined by qRT-PCR and FACS analysis at day 7 of cell culture.

**Figure 1:**
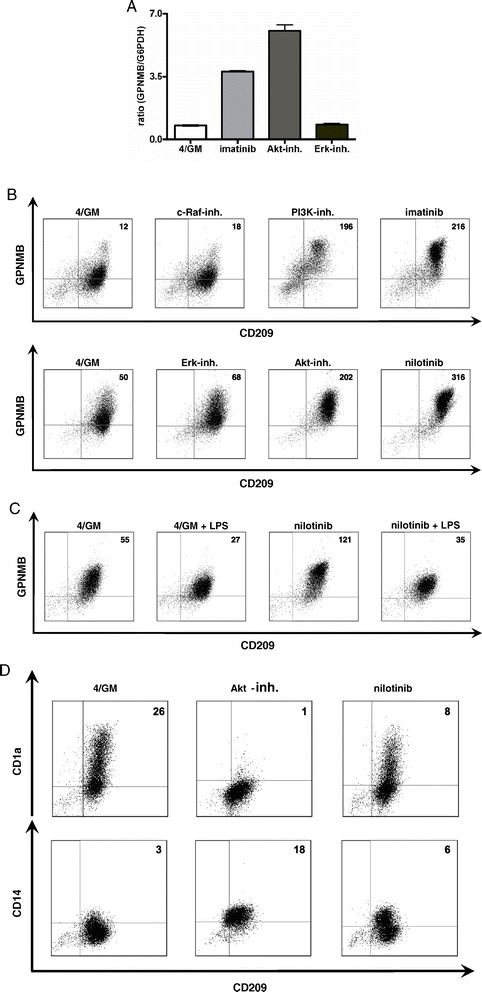
**PI3K/Akt-inhibition upregulates GPNMB gene expression in human moDC.** Immature moDC were generated *in vitro* with GM-CSF and IL-4 alone (4/GM) or with additional TKI (3 μM imatinib or 3 μM nilotinib) or inhibitors of signal transduction (300 nM Akt inhibitor MK2206 (Akt-inh.), 300 nM Erk inhibitor FR180204 (Erk-inh.), 100 nM PI3K inhibitor LY294002 (PI3K-inh.), 20 nM c-Raf inhibitor 553003 (c-Raf-inh.)) and analyzed for GPNMB expression. Exemplary results from at least three independent experiments using different donors are presented. **(A)** qRT-PCR analysis: relative level of GPNMB mRNA. The mean (±SD) of duplicate measurements is shown. **(B, C)** GPNMB protein level of CD209^+^ moDC (of three different donors) was analyzed by flow cytometry. Where indicated, maturation of moDC was induced by LPS. Data were analyzed using FlowJo software and Difference in Median Fluorescence Intensity (DMFI) of CD209^+^ cells is shown in the upper right quadrants. **(D)** Phenotypic changes of immature moDC in the absence (4/GM) or presence of nilotinib or Akt inhibitor were analyzed by flow cytometry. Double stainings were performed with monoclonal antibodies recognizing CD209, CD1a or CD14. DMFI of CD209^+^ cells is shown in the upper right quadrants.

Consistent with our previous findings, incubation with BCR-ABL TKI imatinib or nilotinib from the first day of culturing resulted in a marked increase of GPNMB steady-state mRNA concentrations (Figure 1A) and cell surface protein (Figure 1B) on CD209^+^ (DC-SIGN^+^) moDC. Interestingly, treatment of cells with 125–1000 nM Akt inhibitor or 500–1000 nM of PI3K inhibitor also led to upregulation of GPNMB expression (Figure 1A, B and Additional file 1: Figure S1). In contrast, inhibition of the Erk-pathway by FR180204, c-Raf inhibitor 553008 or MEK1/2 inhibitors U0126 and PD0325901 did not have any significant effect on GPNMB expression (Figure 1A and B or data not shown). In response to triggering TLR4 signaling by lipopolysaccharide (LPS), Akt is phosphorylated rapidly through PI3K [26]. In accordance with this mechanism and our previous findings [20], stimulation of moDC with LPS resulted in downregulation of GPNMB expression and compensated nilotinib-induced upregulation of GPNMB cell surface protein (Figure 1C).

Immunophenotyping using flow cytometry (FACS) revealed that moDC treated with nilotinib or Akt inhibitor consistently retained a more CD14^+^ phenotype and exhibited reduced expression of the DC marker CD1a as compared to untreated cells (Figure 1D) indicating inhibition of full cellular differentiation. Other typical surface markers necessary for T cell activation, such as CD80, CD86 or the DC-specific adhesion receptor DC-SIGN (CD209), were not consistently affected by the different treatments (data not shown). Administration of 300 nM Akt inhibitor slightly increased the percentage of dead cells by an average of 3.5% in comparison with untreated cells (data not shown).

Combined, these experiments demonstrate the functional involvement of the PI3K/Akt pathway in the regulation of the expression of the inhibitory molecule GPNMB in moDC.

### The BCR-ABL TKI imatinib and nilotinib or IL-10 inhibit phosphorylation of Akt in moDC

Recently, we showed that imatinib affects phenotype, cytokine secretion, and T cell stimulatory capacity of moDC due to the inhibition of NF-κB and Akt signaling pathways [27]. To analyze the relevance of PI3K/Akt signaling for the regulation of GPNMB expression, we examined the protein levels of Akt as well as its phosphorylation status in moDC by western blotting. To this end, moDC were generated from different donors, in the presence of imatinib, nilotinib, Akt inhibitor MK2206 or the immunosuppressive cytokine IL-10 as a positive control. GM-CSF and IL-4 activate the PI3K/Akt signaling pathway in monocytes which is critical for differentiation and generation of immature moDC [28]. To keep this pathway active, CD209^+^ purified cells were further incubated for 20 and/or 40 min under the same cell culture conditions as on day 0 to restore the initial conditions prior to lysis.

**Figure 2:**
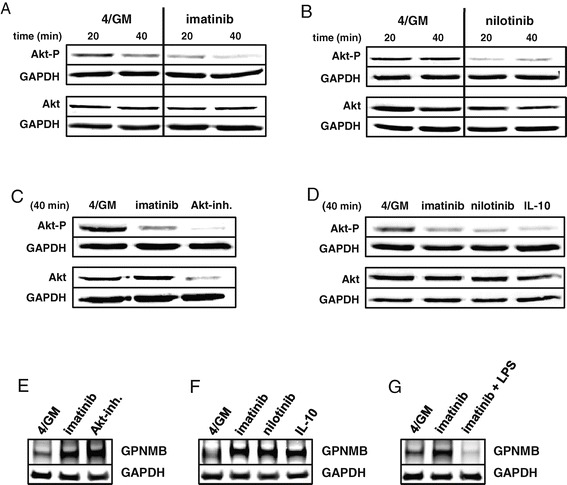
**The BCR-ABL TKI imatinib and nilotinib or IL-10 inhibit phosphorylation of Akt in human moDC.** Western blot analysis of total Akt levels and its phosphorylated form in purified immature CD209^+^ moDC (of four different donors). moDC were generated *in vitro* with GM-CSF and IL-4 alone (4/GM) or with additional **(A)** imatinib (3 μM), **(B)** nilotinib (3 μM), **(C)** Akt inhibitor MK2206 (Akt-inh., 300 nM) or **(D)** IL-10 (10 ng/mL). Indicated time refers to further treatment of cells prior to cell lysis (see Methods). **(E-G)** GPNMB protein levels in moDC were analyzed by western blotting. GAPDH served as loading control. Exemplary results from at least three independent experiments using different donors are presented.

Incubation with imatinib (Figure 2A) or nilotinib (Figure 2B) resulted in decreased amounts of phosphorylated Akt as compared to the respective untreated controls within the indicated timeframe, while the levels of total Akt remained unchanged. As shown in Figure 2C MK2206 (Akt-inh., 300 nM) very effectively inhibited Akt phosphorylation after 40 min in moDC. These findings were approved for IL-10 and TKI with moDC generated from blood monocytes of an additional donor (Figure 2D and Additional file 2: Figure S2). Furthermore, western blot analysis confirmed the FACS data (Figure 1B, C) and revealed a pronounced increase of GPNMB protein levels upon treatment with imatinib or Akt-inhibitor MK2206 (Figure 2E) as well as nilotinib or IL-10 (Figure 2F). As shown in Figure 2G, stimulation of moDC with LPS efficiently compensated imatinib-induced upregulation of GPNMB.

In line with our previous findings these experiments indicate that BCR-ABL TKI and the immunosuppressive cytokine IL-10 concordantly inhibit the phosphorylation of Akt in immature moDC and suggest that Akt dephosphorylation is critically involved in the upregulation of the inhibitory receptor GPNMB.

### Imatinib, nilotinib, Akt inhibitor or IL-10 prevent phosphorylation of GSK3ß in moDC

**Figure 3:**
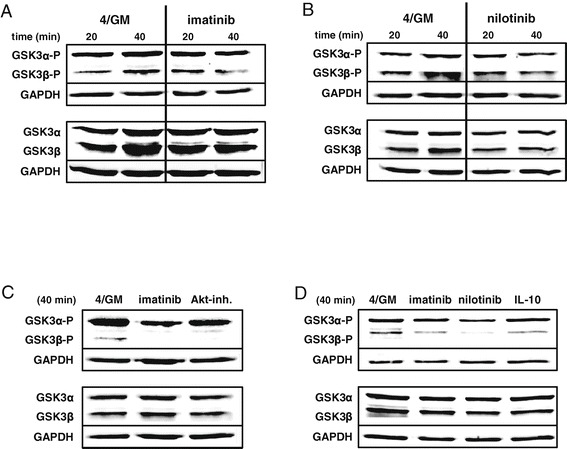
**Imatinib, nilotinib, IL-10 or Akt inhibitor prevent phosphorylation of GSK3ß in human moDC.** Western blot analysis of total GSK3ß and GSK3α, as well as their phosphorylated forms in purified immature CD209^+^ moDC (of four different donors). moDC were generated *in vitro* with GM-CSF and IL-4 alone (4/GM) or with additional **(A)** imatinib (3 μM), **(B)** nilotinib (3 μM), **(C)** Akt inhibitor MK2206 (Akt-inh., 300 nM) or **(D)** IL-10 (10 ng/mL). Indicated time refers to further treatment of cells prior to cell lysis (see Methods). GAPDH served as loading control. Exemplary results from at least three independent experiments using different donors are presented.

A central issue of our study was to elucidate the molecular switch that facilitates transcriptional activation upon BCR-ABL TKI- or IL-10-mediated inhibition of cellular signaling in moDC. Accordingly, we focused our further analyses on proteins that promote gene transcription upon inhibition by Akt. Such a downstream molecule of the PI3K/Akt pathway is the serine/threonine protein kinase GSK3β that phosphorylates a broad range of substrates, including several transcription factors. When the PI3K/Akt pathway is active, GSK3β is inhibited as a result of Akt phosphorylation. Conversely, inhibition of PI3K/Akt signaling results in dephosphorylation and activation of GSK3β [29,30]. Therefore, we next investigated the possibility that the inhibition of Akt by TKI or IL-10 results in dephosphorylation and thereby activation of GSK3β in moDC. For the respective western blot analysis a monoclonal antibody that detects both isoforms of GSK3 (α, β) was used. Consistent with the hypothesized mechanism, we detected substantially lower amounts of phosphorylated GSK3β (upper panels, lower band, 46 kDa) in the samples treated with imatinib (Figure 3A), nilotinib (Figure 3B) or Akt inhibitor MK2206 (Figure 3C) as compared to untreated controls. These experiments were repeated for IL-10 and TKI with moDC generated from blood monocytes of an additional donor (Figure 3D and Additional file 3: Figure S3). The phosphorylation status of GSK3α (Figure 3A-D: upper bands, 51 kDa) as well as the level of unphosphorylated GSK3β (Figure 3A-D: lower panels) was not affected by the treatment of moDC.

These experiments indicate that inhibition of the Akt signaling pathway by BCR-ABL TKI, MK2206 or IL-10 in developing human moDC results in reduced phosphorylation and subsequent activation of GSK3β and suggest a mechanism, by which inhibition of cell signaling could induce transcriptional activation of immune inhibitory molecules such as GPNMB.

### The transcription factor MITF is expressed in progenitor cells, leucocytes and primary moDC

**Figure 4:**
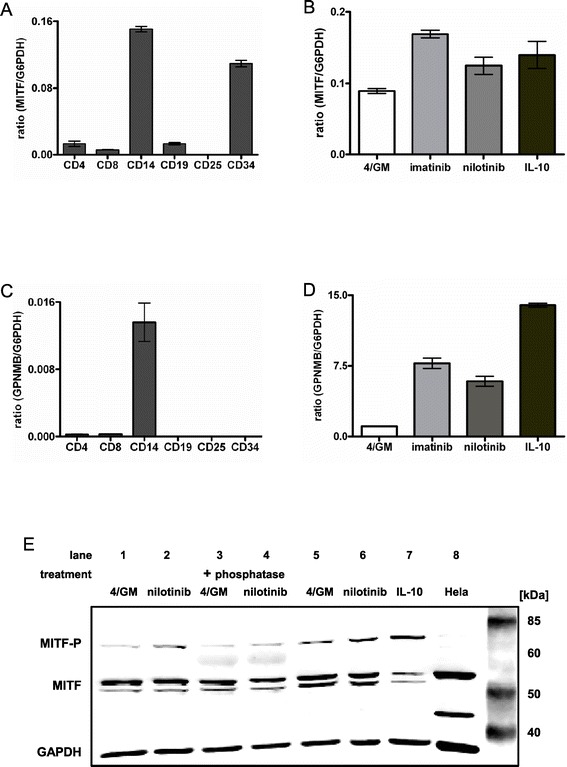
**The transcription factor MITF is expressed in progenitor cells, leucocytes and primary moDC.** Immature moDC were generated *in vitro* with GM-CSF and IL-4 alone (4/GM) or with additional TKI (3 μM imatinib or 3 μM nilotinib) or IL-10 (10 ng/mL). For the analysis of CD34^+^ progenitor and blood cells, cell-type specific total RNA was used. qRT-PCR analysis: **(A, B)** relative level of MITF and **(C, D)** GPNMB mRNA. The mean (±SD) of duplicate measurements is shown. **(E)** MITF protein level and phosphorylation status was analyzed by western blotting in two different donors (lanes 1–4 and lanes 5–7, respectively). Phosphorylated MITF was detected by mobility shift (slower migrating band at 70 kDa). Western blotting revealed an additional, slower migrating band that supposedly represents the phosphorylated protein of higher molecular weight of approximately 70 kDa. “+ phosphatase”: cell lysates were incubated with phosphatase. GAPDH served as loading control. Exemplary results from at least three independent experiments using different donors are presented.

Next we aimed to identify the responsible transcription factor downstream of the PI3K/Akt signaling cascade which is activated by GSK3β. Interestingly, GPNMB expression in melanoblasts and osteoclasts was shown to be dependent on MITF, and in human glioblastoma cells this transcription factor is activated by PI3K/Akt and GSK3β signaling [31,32]. However, its expression pattern and functional role in hematopoietic and blood cells is so far unknown. Therefore, we first examined the expression of MITF mRNA in hematopoietic and blood cells by qRT-PCR. Significant expression was observed only in CD14^+^ monocytes and CD34^+^ progenitor cells. CD4^+^, CD8^+^ and CD19^+^ cells showed very low and CD4^+^CD25^+^ regulatory T cells no expression of MITF (Figure 4A). In our next set of experiments, we analyzed MITF mRNA levels in immature purified CD209^+^ moDC. As shown in Figure 4B, transcripts were detected in untreated cells as well as in samples treated with TKI or IL-10. Notably, steady-state transcript levels increased upon treatment with imatinib, nilotinib or IL-10 and positively correlated with the GPNMB mRNA expression in these cells (Figure 4D and Additional file 4: Figure S4). Interestingly, among the leucocytes analyzed, GPNMB mRNA was detected only in CD14^+^ monocytes used for *in vitro* generation of moDC (Figure 4C). However, moDC displayed significantly higher levels of expression than CD14^+^ cells (Figure 4D).

Next we determined MITF protein expression in purified CD209^+^ moDC by western blotting. In line with the mRNA expression pattern, we observed MITF protein in all analyzed cell extracts. Multiple bands representing the various isoforms were detected, of which the most prominent migrated at approximately 52 and 56 kDa (Figure 4E). Due to lack of available phosphospecific antibodies for MITF, the phosphorylated and therefore activated form could only be detected by mobility shift. Western blotting revealed an additional, slower migrating band that supposedly represents the phosphorylated protein of higher molecular weight at approximately 70 kDa (Figure 4E).

Thus our data show that the transcription factor MITF is expressed in both CD14^+^ monocytes (the starting cells for the *in vitro* generation of moDC) and primary human immature moDC.

### MITF is phosphorylated in moDC after BCR-ABL TKI or IL-10 treatment

In a previous study GSK3 was found to phosphorylate serine 298 of MITF, thereby enhancing the binding to the tyrosinase promoter [33]. When analyzing MITF protein in whole cell lysates of moDC by western blotting, the additional slower migration band was clearly increased after 20 min in the samples incubated with nilotinib or IL-10 as compared to the untreated control (Figure 4E: lanes 2, 6, 7: upper band, approximately 70 kDa). To confirm that the shift in mobility was due to phosphorylation, cell lysates of moDC generated in the presence or absence of nilotinib were incubated with phosphatase. We found that such treatment reduced the intensity of the 70 kDa band (Figure 4E, lane 4) when compared to untreated samples (Figure 4E, lane 2 and 6), thus confirming that the mobility shift described above was due to phosphorylation of MITF. To further verify the specificity of the anti-MITF antibody, the HeLa cell line that reportedly exhibits a prominent MITF-band of 56 kDa was included as a control (Figure 4E: lane 8).

### Phosphorylated MITF translocates into the nucleus upon treatment of moDC with imatinib, nilotinib, IL-10 or Akt inhibitor

**Figure 5:**
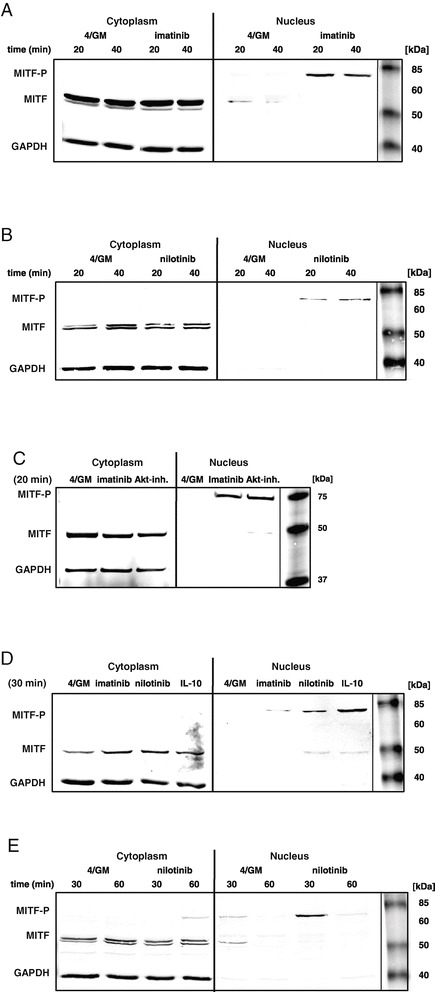
**Upon treatment of moDC with imatinib, nilotinib, IL-10 or MK2206, MITF translocates into the nucleus.** Western blot analysis of MITF level and phosphorylation status in the cytoplasmic or nuclear fraction of purified immature CD209^+^ moDC. moDC were generated *in vitro* with GM-CSF and IL-4 alone (4/GM) or with **(A)** imatinib (3 μM), **(B)** nilotinib (3 μM), **(C)** Akt inhibitor MK2206 (Akt-inh.; 300 nM) or **(D)** IL-10 (10 ng/mL). **(E)** Cells were treated with nilotinib (3 μM). Indicated time refers to further treatment of cells prior to cell lysis (see Methods). GAPDH served as loading control. Exemplary results from at least three independent experiments using different donors are presented.

MITF contains a nuclear localization signal (NLS) and was shown to shuttle between cytoplasmic and nuclear compartments [34]. Therefore, we prepared cytoplasmic and nuclear extracts of the differently treated *in vitro* generated moDC and evaluated the localization of MITF. Western blot analyses of nuclear extracts revealed appearance of phosphorylated MITF after 20 to 40 min in cells generated in the presence of imatinib (Figure 5A: right panel), nilotinib (Figure 5B: right panel) or Akt inhibitor MK2206 (Figure 5C: right panel) indicating nuclear translocation in response to these stimuli. These findings were confirmed for IL-10 and TKI with moDC generated from blood monocytes of an additional donor (Figure 5D: right panel). In contrast, in the cytoplasmic fractions of the respective moDC populations, only unphosphorylated protein was detected (Figure 5A-D: left panels).

Active signaling in the nucleus is terminated by dephosphorylation of transcription factors. In our experiments, the phosphorylated MITF band disappeared between 40 and 60 min as shown exemplary for nilotinib treated cells (Figure 5E: right panel).

Taken together, our results show for the first time that the transcription factor MITF is phosphorylated and translocated into the nucleus upon inhibition of the PI3K/Akt signaling cascade by clinically used BCR-ABL TKI or the immunosuppressive cytokine IL-10 in moDC. In the nucleus MITF is supposed to activate gene expression of inhibitory molecules such as GPNMB.

### MITF regulates GPNMB expression in moDC

**Figure 6:**
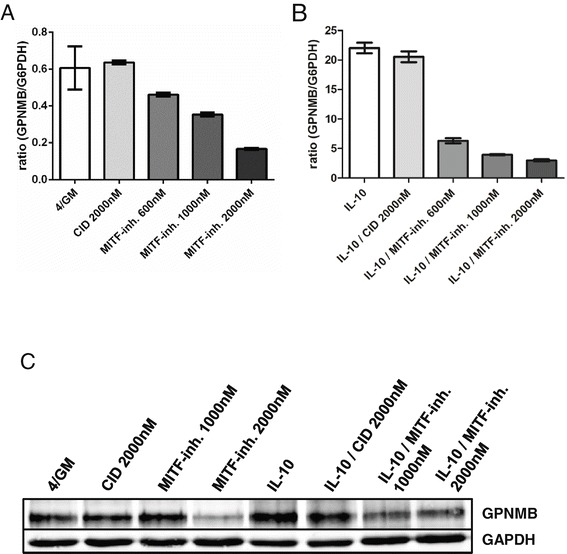
**MITF-Inhibition decreases GPNMB gene expression in moDC.** moDC were generated *in vitro* with GM-CSF, IL-4 and DMSO alone (4/GM) with or without IL-10 and additional MITF inhibitor ML329 (MITF-inh.; 600 nM - 2000 nM) or KLF5 expression inhibitor CID (2000 nM) as control and analyzed for GPNMB expression. **(A, B)** qRT-PCR analysis: relative level of GPNMB mRNA. The mean (±SD) of duplicate measurements is shown. **(C)** GPNMB protein levels were analyzed by western blotting. GAPDH served as loading control. Exemplary results from at least three independent experiments using different donors are presented.

To directly verify the existence of a functional link between MITF and GPNMB expression in moDC, we used the small molecule inhibitor of MITF activity ML329 [22]. To that end, moDC were generated with or without IL-10 in the presence of ML329 or the solvent DMSO alone or CID-5951923 (KLF5 inhibitor) as controls. Importantly, treatment of cells with up to 2000 nM ML329 did neither alter their typical phenotype nor induce apoptosis as analyzed by immune phenotyping and Annexin-V/PI staining (data not shown). As shown in Figure 6A (and Additional file 5: Figure S5), incubation with increasing amounts of ML329 led to a gradual decline of the basal GPNMB mRNA levels to an average of ~30% with 2000 nM ML329 as compared to the DMSO or CID-5951923 control. Remarkably, even in the samples treated with IL-10, where GPNMB was strongly upregulated, mRNA levels were significantly reduced to an average of ~13% with 2000 nM ML329 (Figure 6B and Additional file 5: Figure S5). Decrease of GPNMB at the protein level was confirmed by western blot analyses for moDC generated with or without IL-10 and treated with 1000 nM and 2000 nM ML329 (Figure 6C). Moreover, decrease of cell surface protein on moDC generated with imatinib or nilotinib and treated with 2000 nM ML329 was seen in FACS analyses (Additional file 6: Figure S6).

In summary, these experiments demonstrate that GPNMB expression in moDC is mediated by the transcription factor MITF as endpoint of the (inhibited) PI3K/Akt pathway.

### Inhibition of the PI3K/Akt pathway results in reduction of moDC T cell stimulatory capacity that is restored by soluble SD-4

**Figure 7:**
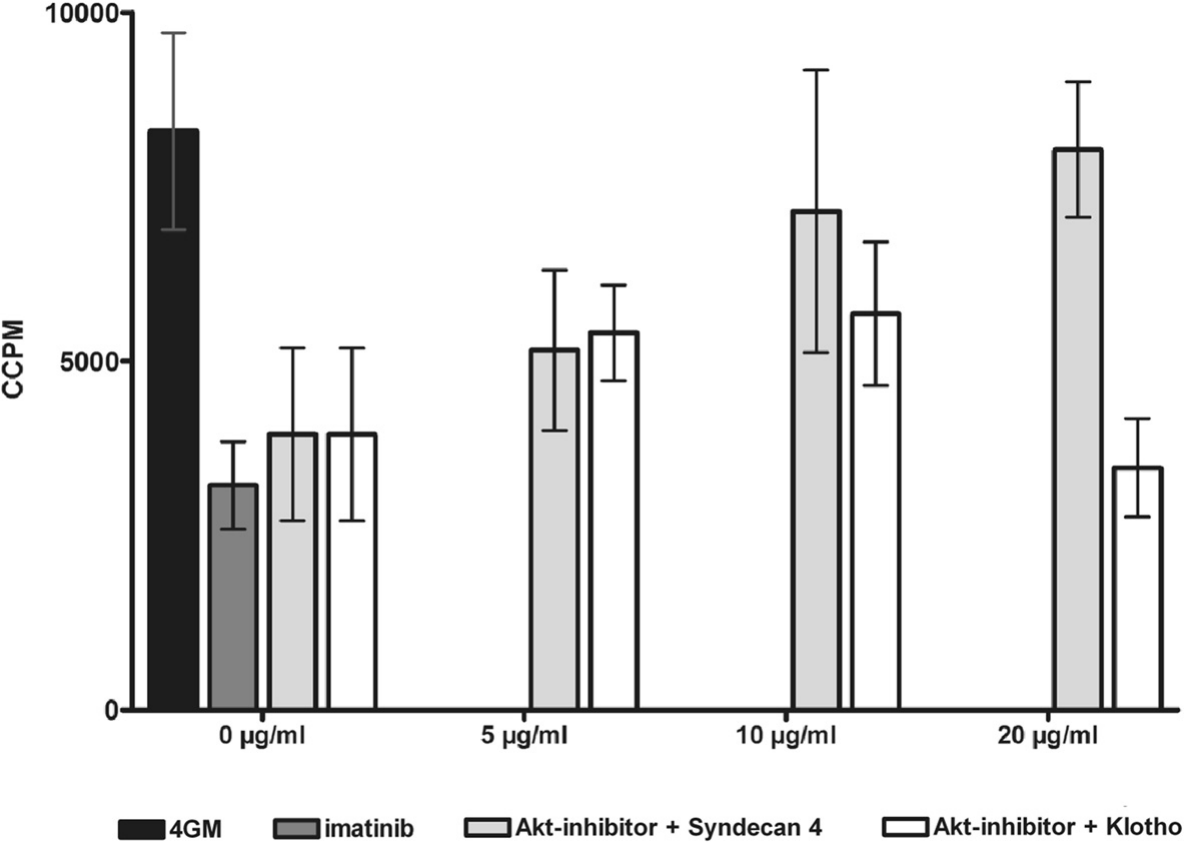
**Akt inhibition reduces the capacity of human moDC to induce T cell responses.** moDC generated *in vitro* with GM-CSF and IL-4 alone (4/GM) or with imatinib (3 μM) or Akt inhibitor MK2206 (300 nM) were used as stimulators in MLR with allogeneic T cells. Increasing concentration (0.0 μg/mL - 20 μg/mL) of blocking soluble recombinant T cell ligand SD-4 were added with recombinant Klotho β serving as control. T cell proliferation was measured by [^3^H]thymidine incorporation. CCPM = corrected counts per minute. The mean (±SD) of quadruple measurements is shown. Exemplary result from three independent experiments using different donors is presented.

We have previously shown that GPNMB upregulation upon exposure to imatinib, dasatinib or nilotinib results in significantly reduced T cell stimulatory capacity of moDC [20]. To confirm the functional relevance of Akt inhibition, we performed T cell proliferation (MLR) assays. For this reason, immature moDC exposed to MK2206 or imatinib during development were cultured with allogeneic peripheral blood mononuclear cells (PBMC) for 5 days and then incubated with ^3^H-thymidine. Treatment with Akt inhibitor or imatinib resulted in significantly reduced capacity to stimulate proliferation of allogeneic T cells to an average of ~50% or ~60%, respectively. (Figure 7 and Additional file 7: Figure S7). Chung *et al.* previously reported SD-4 to be the T cell ligand through which GPNMB mediates its negative co-regulatory function [15-18]. Therefore, we assessed the specific role of GPNMB upregulation upon MK2206 treatment by addition of recombinant ligand SD-4. As demonstrated in Figure 7, blockade of endogenous SD-4 by addition of increasing amounts of soluble SD-4 restored the capacity of MK2206 treated moDC to stimulate proliferation of T cells, while an irrelevant recombinant protein (Klotho β) had no effect (Figure 7 and Additional file 7: Figure S7).

These experiments confirm that targeted inhibition of Akt signaling tunes the development of primary moDC towards a suppressive phenotype and demonstrate that upregulation of GPNMB is critically involved in the inhibition of DC function.

## Discussion

*Ex vivo* generated and manipulated immunogenic DC are used in anti-cancer vaccines in experimental and clinical studies [35]. On the other hand, so-called tolerogenic DC are considered an interesting alternative to conventional immunosuppressive therapies since it has been proven in animal models that their application prevents rejection of transplanted organs. Currently, tolerogenic DC are also tested in patients with GvHD and in animal models of autoimmune diseases [36-41].

The *in vitro* generation of DC from two specific precursor populations is well established: monocytes and CD34^+^ stem cells. However, the usage of primary moDC is advantageous as they are simple to generate in adequate numbers and capable of inducing potent specific T cell responses [42,43]. Adoptive immunotherapy using this cell type for cancer treatment induces remarkable response rates and in clinical studies has shown to improve survival even in patients suffering from advanced disease [44-48].

Fortunately, in the last decade development of novel targeted therapies have significantly improved treatment of cancer. One fascinating example is the introduction of BCR-ABL TKI to CML treatment. However, discontinuation of imatinib results in a relapse rate in about 50% of patients [49]. Combination of targeted therapy and immunotherapy, with the goal to eradicate minimal residual disease is therefore moving into the focus of interest. Essential for the application of such combinatory approaches certainly is to analyze the influence of the respective TKI on cellular signal transduction in immune cells.

Depending on the micro milieu, DC can be found in distinct differentiation stages: immature, mature or semi-mature. Numerous factors induce and/or regulate DC maturation, of which TLR ligands are the most prominent. TLR signaling leads to phosphorylation of inhibitory IκB proteins by activated IKK and the subsequent release of NF-κB transcription factors, which translocate to the nucleus to induce expression of pro-inflammatory target genes [50]. Ozes *et al.* have demonstrated that Akt, activated by TNF, mediates IKK phosphorylation and subsequent NF-κB activation [51]. Simultaneously, activated Akt inhibits GSK3β by phosphorylation at serine 9. It was proposed, if Akt is inactive, that the p105 precursor of the NF-κB p50 subunit is stabilized through phosphorylation by GSK3β, thus preventing formation of functional active NF-κB [52].

Previously, we have established that exposure of human CD14^+^ peripheral blood monocytes to therapeutic concentrations of imatinib during differentiation into moDC affects their phenotype, cytokine secretion, and T cell stimulatory capacity due to inhibition of NF-κB and Akt signaling pathways [27]. Furthermore, we have demonstrated that IL-10 prevents nuclear translocation, DNA binding and TLR-induced nuclear expression of the NF-κB family members c-Rel and Rel-B as well as IRF-3 and IRF-8 as a result of inhibitory effects on the PI3K pathway [19,53].

In the present study we show for the first time that the concordant inhibition of the PI3K/Akt signaling pathway by the clinically used BCR-ABL TKI imatinib or nilotinib, Akt inhibitor MK2206 or the immunosuppressive cytokine IL-10 activates the downstream serine/threonine protein kinase GSK3ß, and subsequently the transcription factor MITF that is translocated into the cell nucleus. Moreover, treatment of moDC with the small molecule inhibitor of the MITF molecular pathway ML329 [22] reduced the expression of GPNMB at the level of mRNA and protein, indicating that GPNMB expression is in fact facilitated by MITF activation.

Based on these and previous results, we suggest the following general model for balancing activating and inhibitory signals in primary moDC: the central regulatory molecules in signal processing are the serine/threonine kinases Akt and GSK3β. Activating signals such as growth factors or TLR stimulation lead to phosphorylation of Akt that causes phosphorylation and thereby inhibition of GSK3β. Inactive GSK3β permits the formation of functional members of the NF-κB transcription factor family that induce the transcription of proinflammatory genes. By contrast, inhibitory stimuli, such as the anti-inflammatory cytokine IL-10 or BCR-ABL TKI, which suppress Akt signaling, lead to activation of GSK3β and the transcription factor MITF that drives expression of inhibitory molecules such as GPNMB.

Lundberg *et al.* recently analysed the gene expression profiles of commonly used *in vitro* DC models (moDC, CD34^+^-derived Langerhans cells, CD34^+^-derived DC and MUTZ-3 DC) and found MITF to be expressed >2 fold higher in moDC as compared to each of the other *in vitro* generated DC [54]. These results and own expression analyses (Figure 4) support our model in which MITF is a relevant transcription factor in moDC. However, a function of MITF for signal transduction in human peripheral blood DC has still to be elucidated.

A number of studies have also shown the direct influence of imatinib on T lymphocytes *in vitro*. Cwynarski *et al.* found that this TKI inhibited T cell proliferation and reduced the production of IFN-γ [55]. Similar results were obtained by Dietz *et al.*: imatinib inhibited T cell proliferation induced by allogeneic DC [56]. A further study found the expression of the activation markers CD25 and CD69 as well as secretion of IL-2 to be suppressed in activated T cells [57]. Taken together, the direct effects of imatinib on T cells, as well as its indirect, mediated via DC, point to the same direction: the inhibition of T cell function. However, the specific contribution of TKI treated DC *in vivo* still has to be proven and elaborated.

Our research provides an important basis for the *in vitro* manipulation of moDC to induce overexpression of GPNMB for the treatment of exaggerated immune responses. Our results might also be relevant in another context: GPNMB is expressed at higher levels in melanoma and breast cancer [58]. The anti-GPNMB antibody-drug conjugate CR011-vcMMAE (glembatumumab vedotin) thus was tested for the treatment of these tumors in phase I/II-clinical studies [59-61]. In this context, attempts were made to increase GPNMB expression in cell lines by treatment with various therapeutics to enhance the binding of CR011-vcMMAE. Interestingly, imatinib, as described here for primary moDC, induced GPNMB expression in melanoma and glioblastoma cell lines. However, the signaling mechanism was not elucidated [62]. It remains an interesting task to investigate the expression and function of GPNMB in other tumor entities in two respects: On the one hand GPNMB is a potential tumor-associated antigen that could be an attractive target for immunotherapeutic approaches. On the other hand GPNMB represents a molecule that suppresses T cell responses and permits tumor escape. For both aspects, specific manipulation of GPNMB expression could be of clinical use for the development of novel treatment approaches for malignant and autoimmune disease.

## Conclusions

The results of the present study demonstrate that the immunosuppressive cytokine IL-10 and the therapeutically used BCR-ABL TKI imatinib or nilotinib, examined here, concordantly lead to dephosphorylation and thereby activation of the serine/threonine protein kinase GSK3ß via inhibition of PI3K/Akt signaling in human moDC. This leads to phosphorylation and translocation of MITF to the nucleus. MITF is a transcription factor whose function in hematopoietic and blood cells was unknown so far. Using a small molecule inhibitor of MITF activity we confirmed that MITF is a direct positive regulator of GPNMB expression in moDC. Moreover, treatment with BCR-ABL TKI or PI3K/Akt inhibitors resulted in profound upregulation of GPNMB that resulted in reduced stimulatory capacity of moDC in MLR with allogenic T cells. This impairment could be restored by addition of the GPNMB T cell ligand SD-4.

Our data extend the current understanding regarding the molecular mechanisms that balance activating and inhibitory signals in DC. Manipulation of the involved signaling cascades and in particular GPNMB expression/function may constitute a promising strategy in combinatory approaches using BCR-ABL TKI and DC-based immunotherapy and may also allow for manipulation of T cell responses in GvHD.

## Methods

### Generation of monocyte-derived dendritic cells (moDC)

moDC were generated *ex vivo* from CD14^+^ peripheral blood primary monocytes that were either purified by magnetic cell sorting (CD14 MicroBeads, Miltenyi, Bergisch Gladbach, Germany) or plastic adherence. Peripheral blood mononuclear cells (PBMC) were isolated by Ficoll/Paque (Biochrom, Berlin, Germany) density gradient centrifugation of buffy coats obtained from healthy volunteers (Blood Donation Center, University of Tübingen). For plastic adherence cells were seeded (1 × 10^8^/10 mL) into 75 cm^2^ cell culture flasks (Corning, Cambridge, MA, USA) in serum-free X-VIVO 20 medium (Cambrex Bio Science, Verviers, Belgium). After 2 h of incubation at 37°C/5% CO_2_, non-adherent cells were removed. The monocytes were cultured in 10 mL RP10 medium (RPMI 1640 with glutamax-I, supplemented with 10% inactivated fetal calf serum, and antibiotics (Invitrogen, Karlsruhe, Germany)) supplemented with granulocyte macrophage colony-stimulating factor (GM-CSF, 100 ng/mL; Leukine Liquid Sargramostim, Sanofi, Bridgewater, USA) and IL-4 (20 ng/mL; R&D Systems, Wiesbaden, Germany) added every 2^nd^ day for 7 days. IL-10 (10 ng/mL; R&D Systems), imatinib (3 μM; Cayman, Biomol, Hamburg, Germany), nilotinib (3 μM; Cayman), Akt inhibitor MK2206 (300 nM; Selleckchem, München, Germany), Erk inhibitor FR180204 (300 nM; Calbiochem, Merck Millipore, Darmstadt, Germany), PI3K inhibitor LY294002 (100 nM; Cayla - InvivoGen, Toulouse, France), c-Raf inhibitor 553003 (20 nM; Calbiochem)), MITF inhibitor ML329 (200 nM - 2000 nM in dimethyl sulfoxide (DMSO); Glixx Laboratories, Southborough, MA, USA) or Kruppel-like factor 5 (KLF5) expression inhibitor CID-5951923 (2000 nM in DMSO; Glixx Laboratories) were added starting from day 0 of cell culture every 2^nd^ day where indicated. Maturation was induced on day 6 by adding LPS (TLR4L, 100 ng/mL; Sigma-Aldrich, Deisenhofen, Germany) where indicated. After 7 days of culture, if necessary, DC-SIGN^+^ (CD209^+^) moDC were enriched to > 90% purity prior to qRT-PCR and western blot analyses (CD209 MicroBead Kit, Miltenyi). Prior to lysis, purified cells were further incubated for 20, 40, 60 or 90 min under the conditions that were applied at the beginning of culture.

### Quantitative reverse transcriptase PCR (qRT-PCR)

Quantification of GPNMB gene transcripts was conducted using a LightCycler carousel-based system (Roche, Mannheim, Germany) as described previously [20]. MITF transcripts (all isoforms) were quantified with primers 5’-ggagcttccaaaacaagcag-3’, 5’-acaagtgtgctccgtctcttc-3’ and Universal ProbeLibrary probe #68 (Roche). The relative mRNA levels were calculated as the ratio target gene/G6PDH. For the analysis of hematopoietic and blood cells, cell-type specific total RNA (Miltenyi) was used.

### Immunostaining

moDC were stained using FITC-, PE-, PerCP-Cy5.5 or allophycocyanin (APC) conjugated mouse monoclonal antibodies against CD1a, CD86, CD14 (PharMingen, Hamburg, Germany), CD80, CD83, HLA-DR, (Becton Dickinson, Heidelberg, Germany), CD86 (PharMingen), DC-SIGN (CD209; eBioscience, Frankfurt, Germany), and mouse IgG isotype control (Becton Dickinson, eBioscience). GPNMB was detected using an anti-human GPNMB antibody (R&D Systems) conjugated to PE (Lightning-Link R-PE conjugation kit, Innova Bioscience, Cambrige, UK).

To exclude dead cells a viability dye (Fixable Viability Dye eFluor 660; eBioscience) was included in all analyses. For detection of apoptosis, the Annexin V-Fluos Staining kit (Roche) was used according to the instructions of the manufacturer. Analyses were performed on a FACSCalibur cytometer (Becton Dickinson). Data were analyzed using FlowJo software. The values have been calculated as follows: DFMI = median fluorescence intensity of CD209^+^ cells - median isotype control fluorescence intensity of CD209^+^ cells. Histogram overlays are displayed as %Max, scaling each curve to mode = 100%.

### Western blotting

If necessary, prior to lysis, positive selection of DC-SIGN (CD209) expressing cells was conducted using the CD209 (DC-SIGN) MicroBead Kit (Miltenyi). Whole cell lysates were prepared from moDC as described previously [20]. Separation of nuclear extract from the cytoplasmic fraction was performed using the Nuclear/Cytosol Fractionation Kit (BioVision; BioCat, Heidelberg, Germany). To prevent proteolytic degradation during cell lysis, Halt Protease and Phosphatase Inhibitor Cocktail (Fisher Scientific, Schwerte, Germany) was added to the lysis buffer. The protein levels of Akt-(P), GSK3α/β-(P) and MITF were determined by separating 10–30 μg whole cell lysates, nuclear or cytoplasmic protein fractions on a 10.5% or 12.0% SDS-polyacrylamide gel and subsequent transfer of protein to nitrocellulose membranes (Whatman, Dassel, Germany). The blots were probed with the following primary antibodies: monoclonal mouse anti-human Akt (R&D Systems), monoclonal rabbit anti-human Phospho-Akt (Ser473), monoclonal rabbit anti-human GSK3α/β, polyclonal rabbit anti-human Phospho-GSK3α/β (Ser 21/9) (Cell Signaling, Frankfurt, Germany), polyclonal rabbit anti-human MITF (abcam, Cambridge, UK) and polyclonal rabbit-anti-GAPDH (Merck Millipore, Darmstadt, Germany) or monoclonal mouse-anti-GAPDH (R&D Systems). Corresponding secondary antibodies were purchased from LI-COR Biotechnology (Bad Homburg, Germany): IRDye 680 Donkey anti-rabbit IgG (H + L), IRDye 680RD Donkey anti-mouse IgG (H + L), IRDye 800CW Donkey anti-rabbit IgG (H + L) and IRDye 800CW Donkey anti-rabbit IgG (H + L). Different antigens were detected simultaneously on the same blot using IRDye secondary antibodies labeled with spectrally distinct fluorescent dyes. The Odyssey Infrared Imaging System (LI-COR Biotechnology) was used for western blot analysis.

### Mixed lymphocyte reaction (MLR)

moDC were inactivated by γ-radiation at 30 Gy, 100%, and were seeded into 96-well microplates (Greiner Bio-One, Frickenhausen, Germany) at concentrations of 1×10^4^ cells/well. Blocking recombinant ligand SD-4 (R&D Systems) was added with recombinant Klotho β (R&D Systems) serving as control. A total of 1×10^5^ responding cells from freshly isolated allogeneic PBMC were added to the previously prepared 1×10^4^ stimulator cells (moDC). Thymidine incorporation was measured on day 5 by a 16 h pulse with [^3^H]thymidine (0.5 μCi [0.0185 MBq]/well; GE Health- care, Munich, Germany).

### Availability of supporting data

The data sets supporting the results of this article are included within the article and its additional files.

## Additional files

Additional file 1: Figure S1. PI3K/Akt-inhibition upregulates GPNMB mRNA levels in human moDC. Combined analysis of different donors. Immature moDC were generated *in vitro* with GM-CSF and IL-4 alone (4/GM) or with additional Akt inhibitor MK2206 (300 nM) or Erk inhibitor FR180204 (300 nM) and analyzed for GPNMB mRNA expression by qRT-PCR. The relative level of GPNMB mRNA in a sample was expressed as the ratio GPNMB/G6PDH. The values were normalized to 100% for IL-4 and GM-CSF treated moDC. Stars indicate significance (**P < .01, n.s. = not significant; Wilcoxon matched-pairs signed rank test). Additional file 2: Figure S2. Imatinib, nilotinib, Akt inhibitor or IL-10 inhibit phosphorylation of Akt in human moDC. Combined analysis of different donors. moDC were generated *in vitro* with GM-CSF and IL-4 alone (4/GM) or with additional imatinib (3 μM), nilotinib (3 μM), Akt inhibitor MK2206 (300 nM) or IL-10 (10 ng/mL) and analyzed by western blotting. The relative level of phosphorylated Akt protein (Akt-P) in a sample was expressed as the ratio Akt-P/GAPDH (loading control). Quantitative analysis was performed using the LI-COR Odyssey Application Software 3.0. The values were normalized to 100% for IL-4 and GM-CSF treated moDC. The mean (±SD) obtained from measurements of different donors is shown. The raw data were used to perform Student’s *t*-test (ratio paired, two-sided, equal variance). Stars indicate significance (**P < .01, ***P < .003, n.s. = not significant). Additional file 3: Figure S3. Imatinib, nilotonib, Akt inhibitor or IL-10 prevent phosphorylation of GSK3ß in human moDC. Combined analysis of different donors. moDC were generated *in vitro* with GM-CSF and IL-4 alone (4/GM) or with additional imatinib (3 μM), nilotinib (3 μM), Akt inhibitor MK2206 (300 nM) or IL-10 (10 ng/mL) and analyzed by western blotting. The relative level of phosphorylated GSK3ß protein (GSK3ß-P) in a sample was expressed as the ratio GSK3ß-P/GAPDH (loading control). Quantitative analysis was performed using the LI-COR Odyssey Application Software 3.0. The values were normalized to 100% for IL-4 and GM-CSF treated moDC. The mean (±SD) obtained from measurements of different donors is shown. The raw data were used to perform Student’s *t*-test (ratio paired, two-sided, equal variance). Stars indicate significance (*P < .05, **P < .01, ***P < .003, n.s. = not significant). Additional file 4: Figure S4. MITF transcript levels are increased upon treatment with imatinib, nilotinib or IL-10. Combined analysis of different donors. Immature moDC were generated *in vitro* with GM-CSF and IL-4 alone (4/GM) or with additional TKI (3 μM imatinib or 3 μM nilotinib) or IL-10 and analyzed for MITF mRNA expression by qRT-PCR. The relative level of MITF mRNA in a sample was expressed as the ratio MITF/G6PDH. The values were normalized to 100% for IL-4 and GM-CSF treated moDC. Additional file 5: Figure S5. MITF-Inhibition downregulates GPNMB mRNA expression in moDC. Combined analysis of different donors. Analysis of GPNMB mRNA levels by qRT-PCR. moDC were generated *in vitro* with **(A)** GM-CSF, IL-4 and DMSO alone (4/GM) or with additional MITF inhibitor ML329 (MITF-inh.; 200 nM - 2000 nM) or KLF5 expression inhibitor CID (2000 nM) as control. **(B)** moDC were generated with additional IL-10 alone or together with MITF inhibitor ML329 (IL-10; MITF-inh.; 200 nM - 2000 nM) or KLF5 expression inhibitor CID (IL-10; CID 2000 nM) as control. The relative level of GPNMB mRNA in a sample was expressed as the ratio GPNMB/G6PDH. The values were normalized to 100% for DMSO or DMSO/IL-10 treated moDC. The raw data were used to perform Student’s *t*-test (ratio paired, two-sided, equal variance). Stars indicate significance (*P < .05, **P < .01, ***P < .003, n.s. = not significant). Additional file 6: Figure S6. MITF-Inhibition decreases GPNMB cell surface protein on moDC generated with imatinib or nilotinib. moDC were generated *in vitro* with GM-CSF, IL-4 and imatinib or nilotinib alone and additional MITF inhibitor ML329 (MITF-inh.; 2000 nM) or KLF5 expression inhibitor CID (2000 nM) as control. GPNMB protein level of CD209^+^ moDC was analyzed by flow cytometry. Data were analyzed using FlowJo software and histogram overlays are displayed as %Max, scaling each curve to mode = 100%. Additional file 7: Figure S7. Akt inhibition reduces the capacity of human moDC to induce T cell responses. Combined analysis of different donors. moDC generated *in vitro* with GM-CSF and IL-4 alone (4/GM) or with imatinib (3 μM) or Akt inhibitor MK2206 (Akt.-inh., 300 nM) were used as stimulators in MLR with allogeneic T cells. T cell proliferation was measured by [^3^H]thymidine incorporation. CCPM = corrected counts per minute. **(A)** Combined analysis of 5 different donors. The values were normalized to 100% for IL-4 and GM-CSF treated moDC. **(B)** Increasing concentration (0.0 μg/mL - 20.0 μg/mL) of blocking soluble recombinant T cell ligand SD-4 were added with recombinant Klotho β serving as control. Combined analyses of 3 different donors. The values were normalized to 100% for Akt inhibitor MK2206 (Akt.-inh., 300 nM) treated moDC. The raw data were used to perform Student’s *t*-test (ratio paired, two-sided, equal variance). Stars indicate significance (*P < .05, n.s. = not significant; absolute data were analyzed using Student’s *t*-test (ratio paired, one-sided, equal variance).

## Abbreviations

APC: Antigen-presenting cells
CML: Chronic myeloid leukemia
DC: Dendritic cell(s)
DC-SIGN: DC-specific ICAM-3 grabbing nonintegrin
DMSO: Dimethyl sulfoxide
FACS: Flow Cytometry
FITC: Fluorescein isothiocyanate
GM-CSF: Granulocyte macrophage colony-stimulating factor
GPNMB: Glycoprotein NMB
GSK3: Glycogen synthase kinase-3
GvHD: Graft-versus-host disease
IKK: IκB kinase
LPS: Lipopolysaccharide
MITF: Microphthalmia-associated transcription factor
MLR: Mixed lymphocyte reactions
moDC: monocyte-derived dendritic cells
NF-κB: nuclear factor-κB
PBMC: Peripheral blood mononuclear cells
PDGF: Platelet-derived growth factor
PE: Phycoerythrin
PerCP: Peridinin-chlorophyll proteins
PI3K: Phosphatidylinositide 3-kinase
qRT-PCR: Quantitative reverse transcriptase PCR
SD-4: Syndecan-4
TKI: Tyrosine kinase inhibitors
TLR: Toll-Like Receptor
