# Erratum to: The transcription factor MITF is a critical regulator of GPNMB expression in dendritic cells

**DOI:** 10.1186/s12964-016-0134-1

**Published:** 2016-05-17

**Authors:** Michael Gutknecht, Julian Geiger, Simone Joas, Daniela Dörfel, Helmut R. Salih, Martin R. Müller, Frank Grünebach, Susanne M. Rittig

**Affiliations:** Department of Internal Medicine II, Oncology, Hematology, Immunology, Rheumatology and Pulmology, University of Tübingen, Otfried-Müller-Str. 10, Tübingen, 72076 Germany

After publication of this work [1], we noted that we inadvertently missed to include research funding organisations that supported this work. Missing research funding was supplemented in the acknowledgements.
