# Supplementary figures and images for: The transcription factor MITF is a critical regulator of GPNMB expression in dendritic cells
Source: Cell Commun Signal. 2015 Mar 24;13:19. doi: 10.1186/s12964-015-0099-5 (PMC4422548; doi:10.1186/s12964-015-0099-5)

## Slide 1
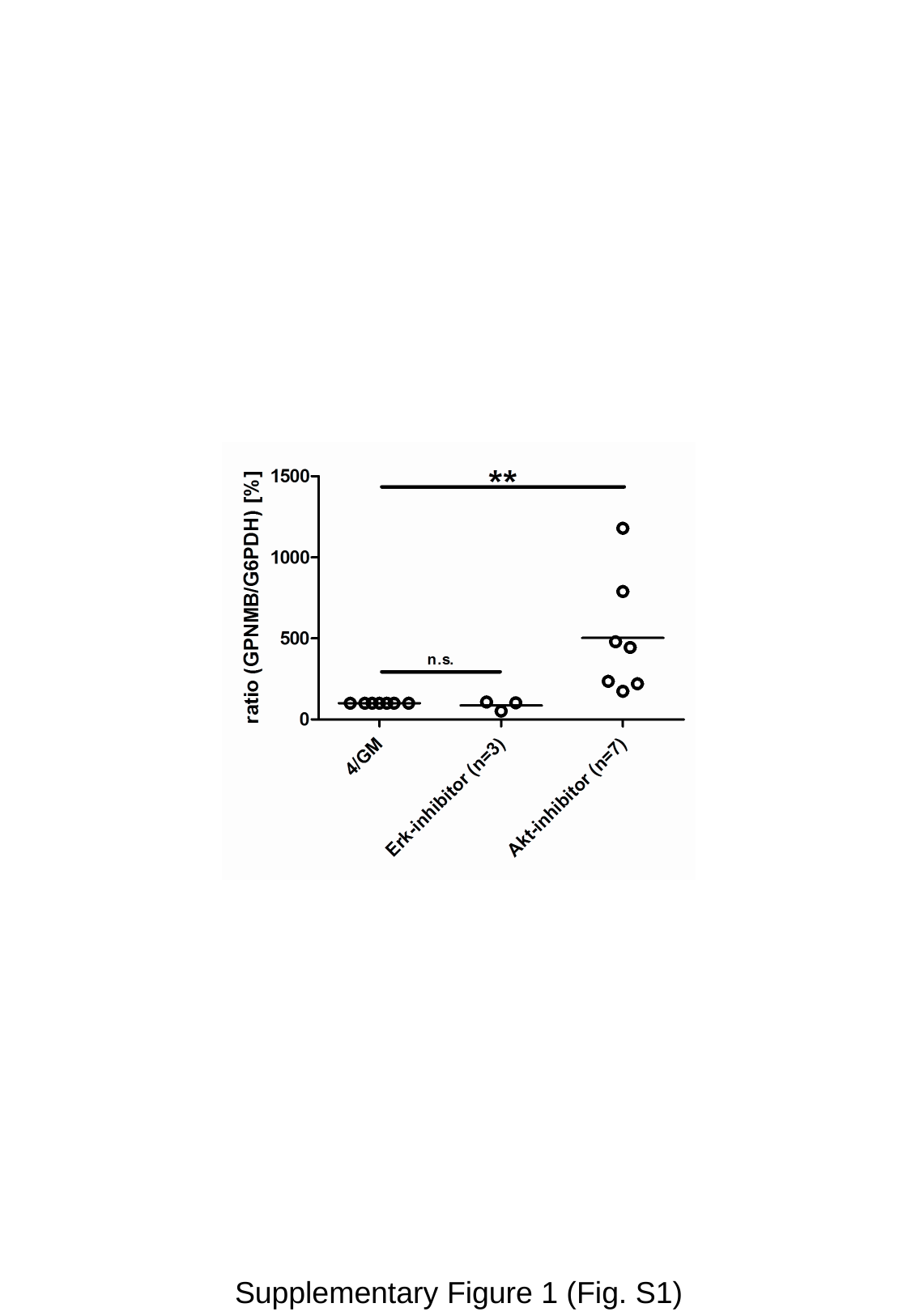

Supplementary Figure 1 (Fig. S1)

Supplement: Additional file 1: Figure S1. — PI3K/Akt-inhibition upregulates GPNMB mRNA levels in human moDC. Combined analysis of different donors. Immature moDC were generated in vitro with GM-CSF and IL-4 alone (4/GM) or with additional Akt inhibitor MK2206 (300 nM) or Erk inhibitor FR180204 (300 nM) and analyzed for GPNMB mRNA expression by qRT-PCR. The relative level of GPNMB mRNA in a sample was expressed as the ratio GPNMB/G6PDH. The values were normalized to 100% for IL-4 and GM-CSF treated moDC. Stars indicate significance (**P < .01, n.s. = not significant; Wilcoxon matched-pairs signed rank test). [file 12964_2015_99_MOESM1_ESM.ppt]

## Slide 1
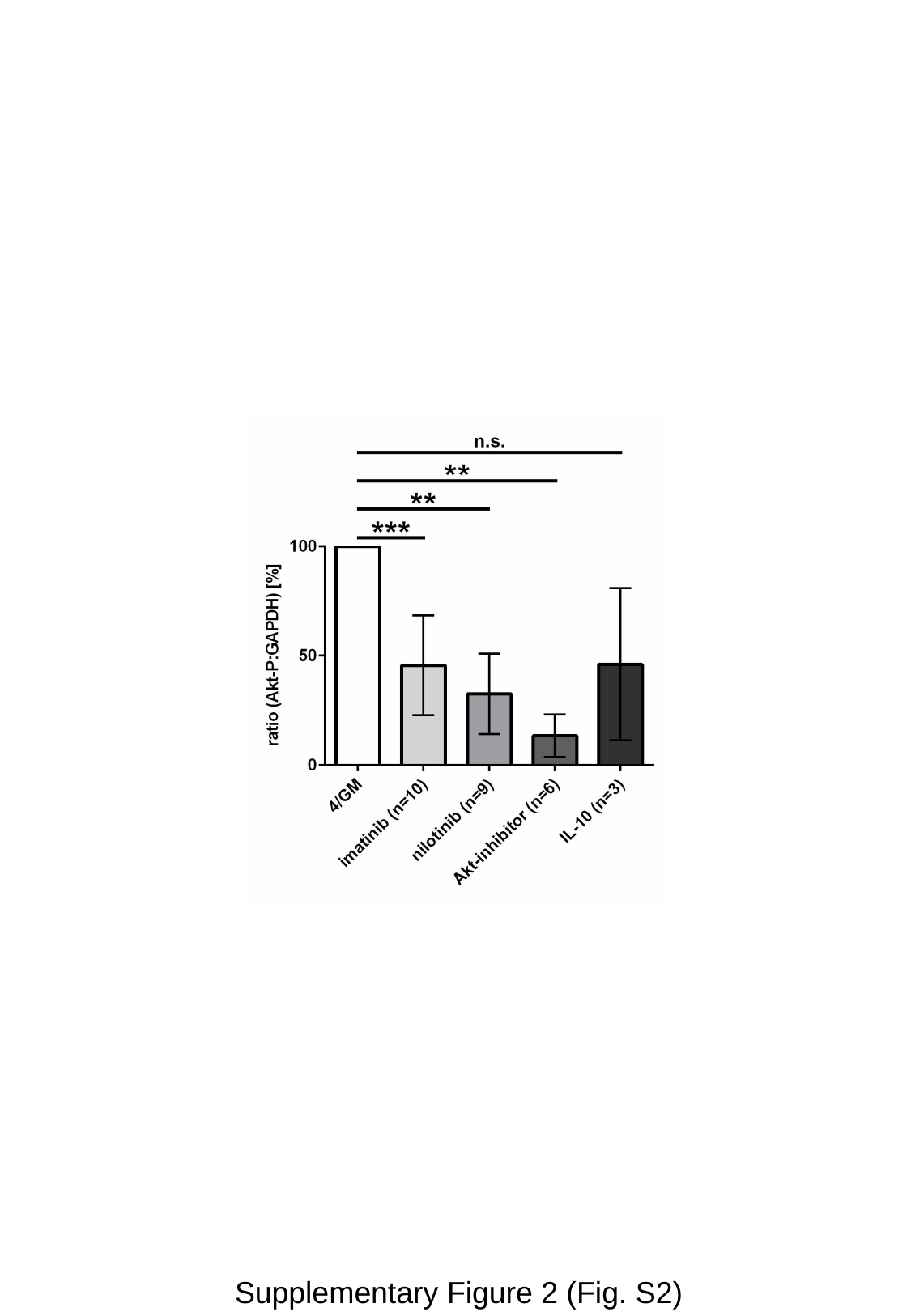

Supplementary Figure 2 (Fig. S2)

Supplement: Additional file 2: Figure S2. — Imatinib, nilotinib, Akt inhibitor or IL-10 inhibit phosphorylation of Akt in human moDC. Combined analysis of different donors. moDC were generated in vitro with GM-CSF and IL-4 alone (4/GM) or with additional imatinib (3 μM), nilotinib (3 μM), Akt inhibitor MK2206 (300 nM) or IL-10 (10 ng/mL) and analyzed by western blotting. The relative level of phosphorylated Akt protein (Akt-P) in a sample was expressed as the ratio Akt-P/GAPDH (loading control). Quantitative analysis was performed using the LI-COR Odyssey Application Software 3.0. The values were normalized to 100% for IL-4 and GM-CSF treated moDC. The mean (±SD) obtained from measurements of different donors is shown. The raw data were used to perform Student’s t-test (ratio paired, two-sided, equal variance). Stars indicate significance (**P < .01, ***P < .003, n.s. = not significant). [file 12964_2015_99_MOESM2_ESM.ppt]

## Slide 1
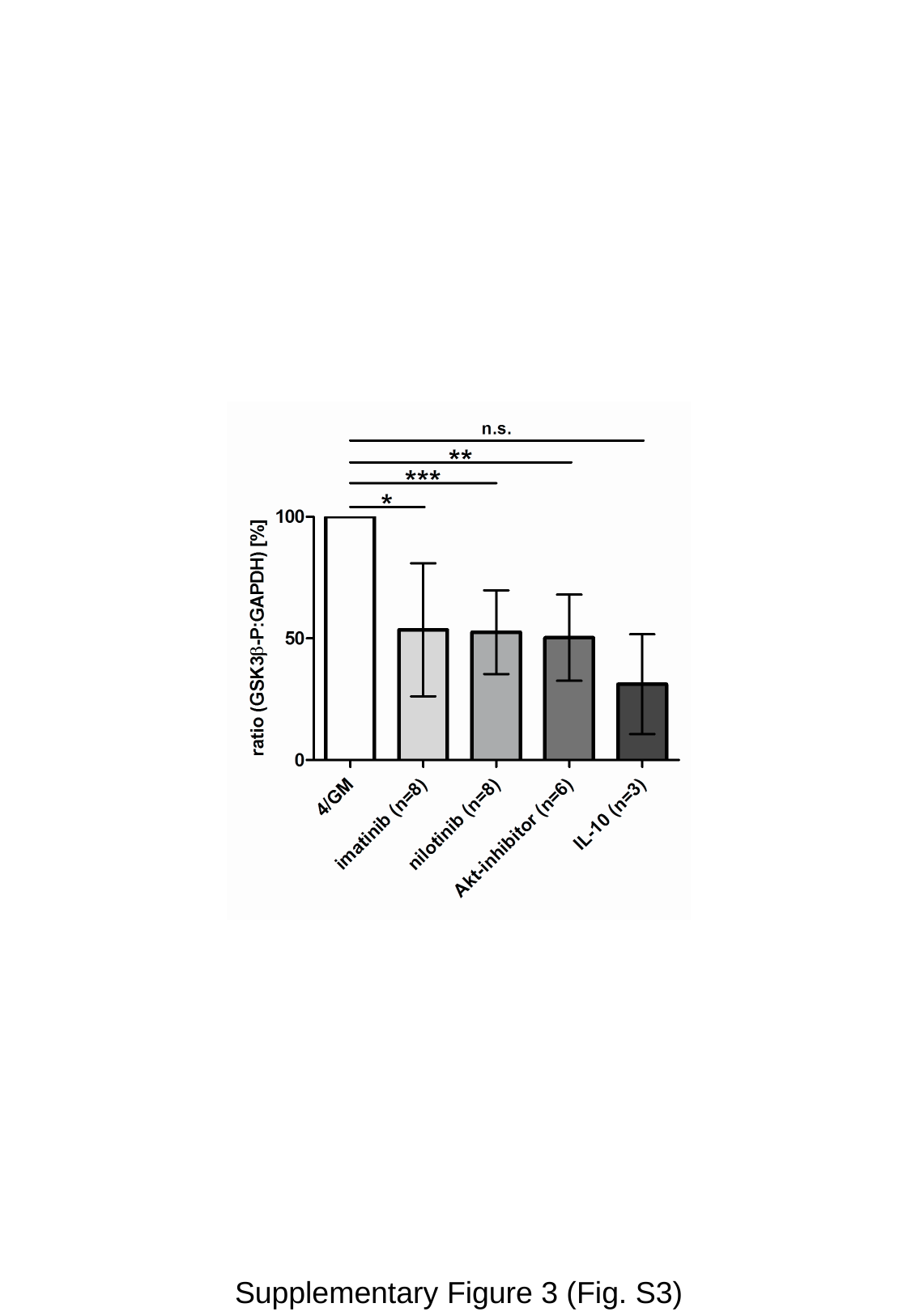

Supplementary Figure 3 (Fig. S3)

Supplement: Additional file 3: Figure S3. — Imatinib, nilotonib, Akt inhibitor or IL-10 prevent phosphorylation of GSK3ß in human moDC. Combined analysis of different donors. moDC were generated in vitro with GM-CSF and IL-4 alone (4/GM) or with additional imatinib (3 μM), nilotinib (3 μM), Akt inhibitor MK2206 (300 nM) or IL-10 (10 ng/mL) and analyzed by western blotting. The relative level of phosphorylated GSK3ß protein (GSK3ß-P) in a sample was expressed as the ratio GSK3ß-P/GAPDH (loading control). Quantitative analysis was performed using the LI-COR Odyssey Application Software 3.0. The values were normalized to 100% for IL-4 and GM-CSF treated moDC. The mean (±SD) obtained from measurements of different donors is shown. The raw data were used to perform Student’s t-test (ratio paired, two-sided, equal variance). Stars indicate significance (*P < .05, **P < .01, ***P < .003, n.s. = not significant). [file 12964_2015_99_MOESM3_ESM.ppt]

## Slide 1
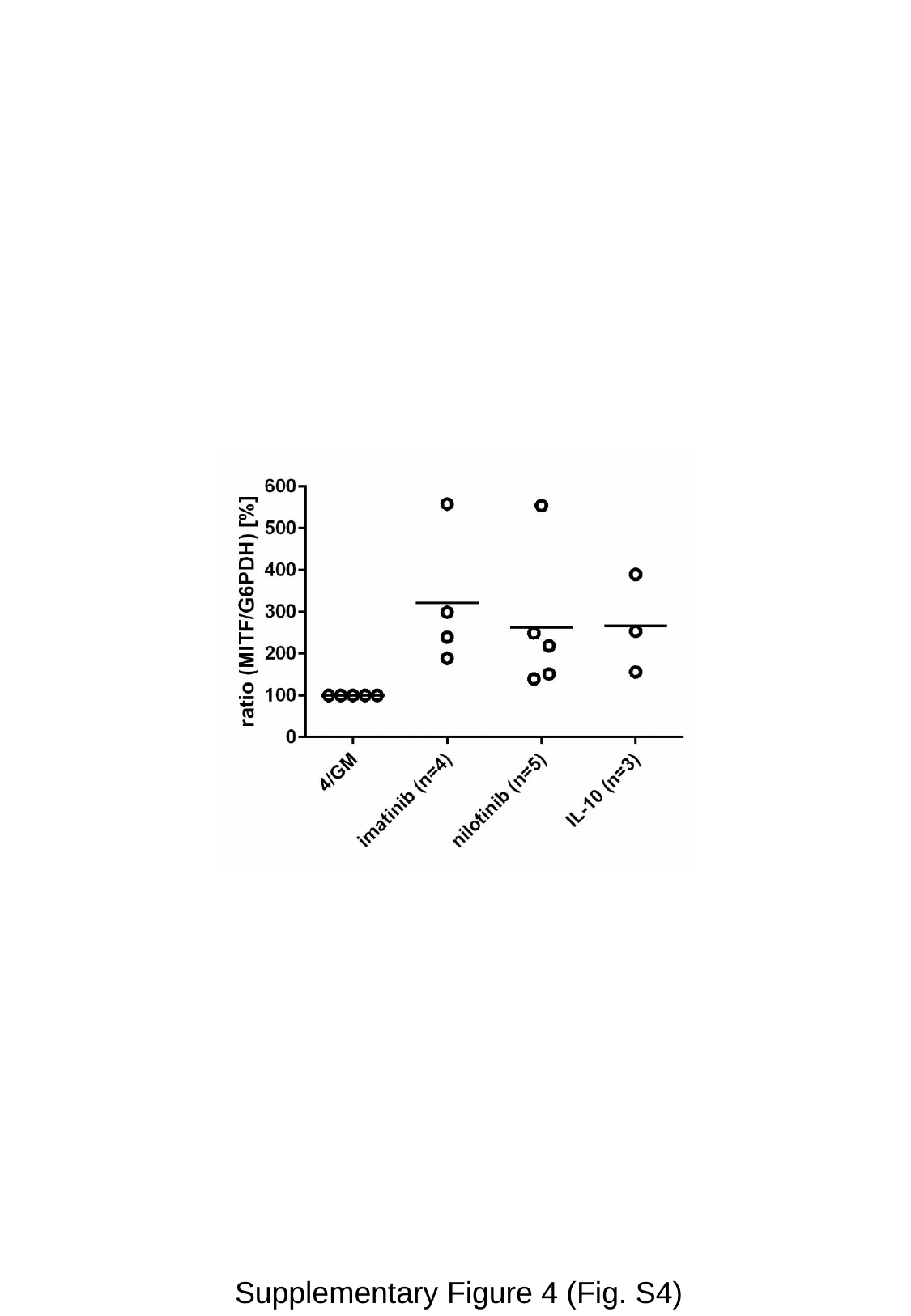

Supplementary Figure 4 (Fig. S4)

Supplement: Additional file 4: Figure S4. — MITF transcript levels are increased upon treatment with imatinib, nilotinib or IL-10. Combined analysis of different donors. Immature moDC were generated in vitro with GM-CSF and IL-4 alone (4/GM) or with additional TKI (3 μM imatinib or 3 μM nilotinib) or IL-10 and analyzed for MITF mRNA expression by qRT-PCR. The relative level of MITF mRNA in a sample was expressed as the ratio MITF/G6PDH. The values were normalized to 100% for IL-4 and GM-CSF treated moDC. [file 12964_2015_99_MOESM4_ESM.ppt]

## Slide 1
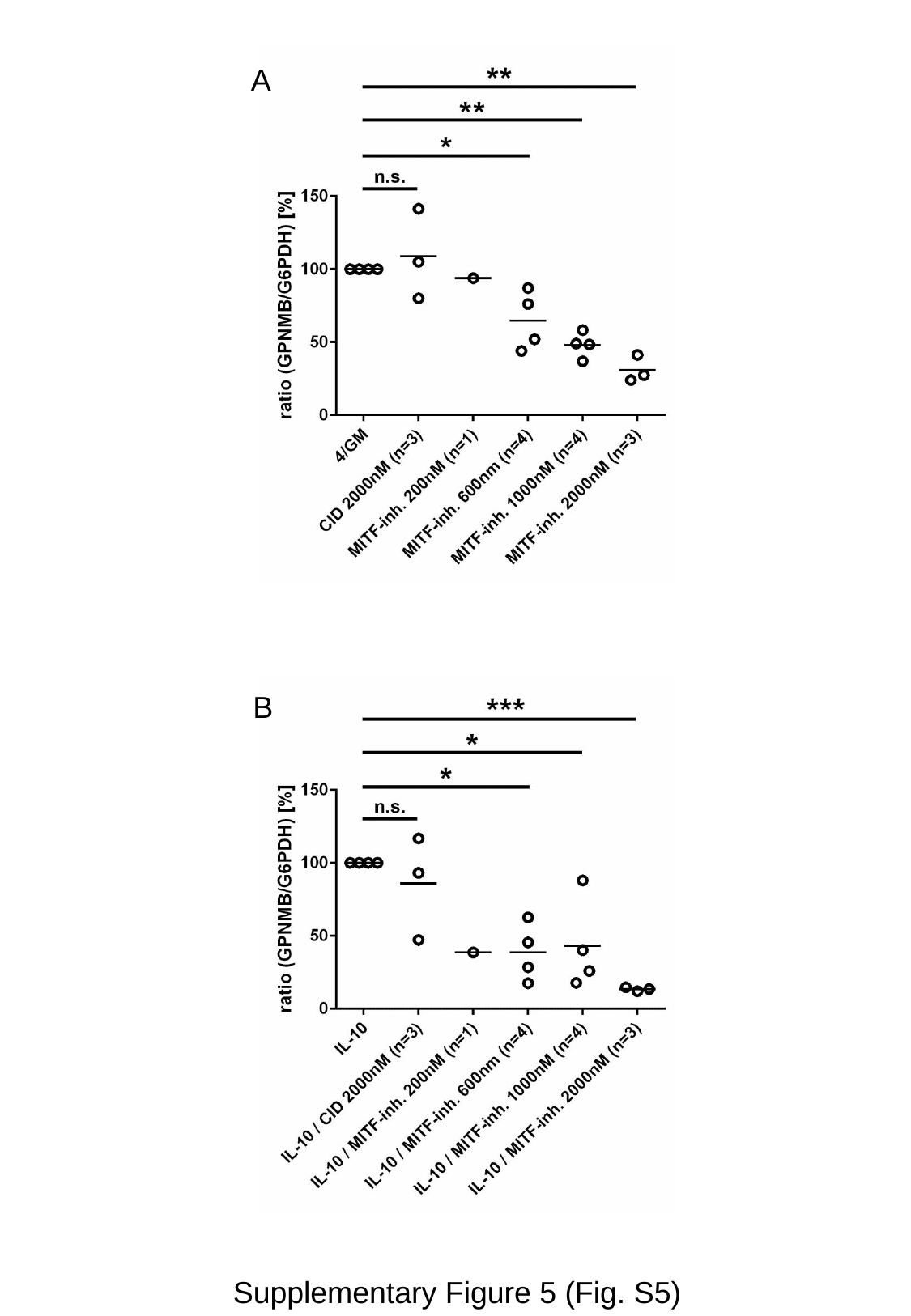

A
B
Supplementary Figure 5 (Fig. S5)

Supplement: Additional file 5: Figure S5. — MITF-Inhibition downregulates GPNMB mRNA expression in moDC. Combined analysis of different donors. Analysis of GPNMB mRNA levels by qRT-PCR. moDC were generated in vitro with (A) GM-CSF, IL-4 and DMSO alone (4/GM) or with additional MITF inhibitor ML329 (MITF-inh.; 200 nM - 2000 nM) or KLF5 expression inhibitor CID (2000 nM) as control. (B) moDC were generated with additional IL-10 alone or together with MITF inhibitor ML329 (IL-10; MITF-inh.; 200 nM - 2000 nM) or KLF5 expression inhibitor CID (IL-10; CID 2000 nM) as control. The relative level of GPNMB mRNA in a sample was expressed as the ratio GPNMB/G6PDH. The values were normalized to 100% for DMSO or DMSO/IL-10 treated moDC. The raw data were used to perform Student’s t-test (ratio paired, two-sided, equal variance). Stars indicate significance (*P < .05, **P < .01, ***P < .003, n.s. = not significant). [file 12964_2015_99_MOESM5_ESM.ppt]

## Slide 1
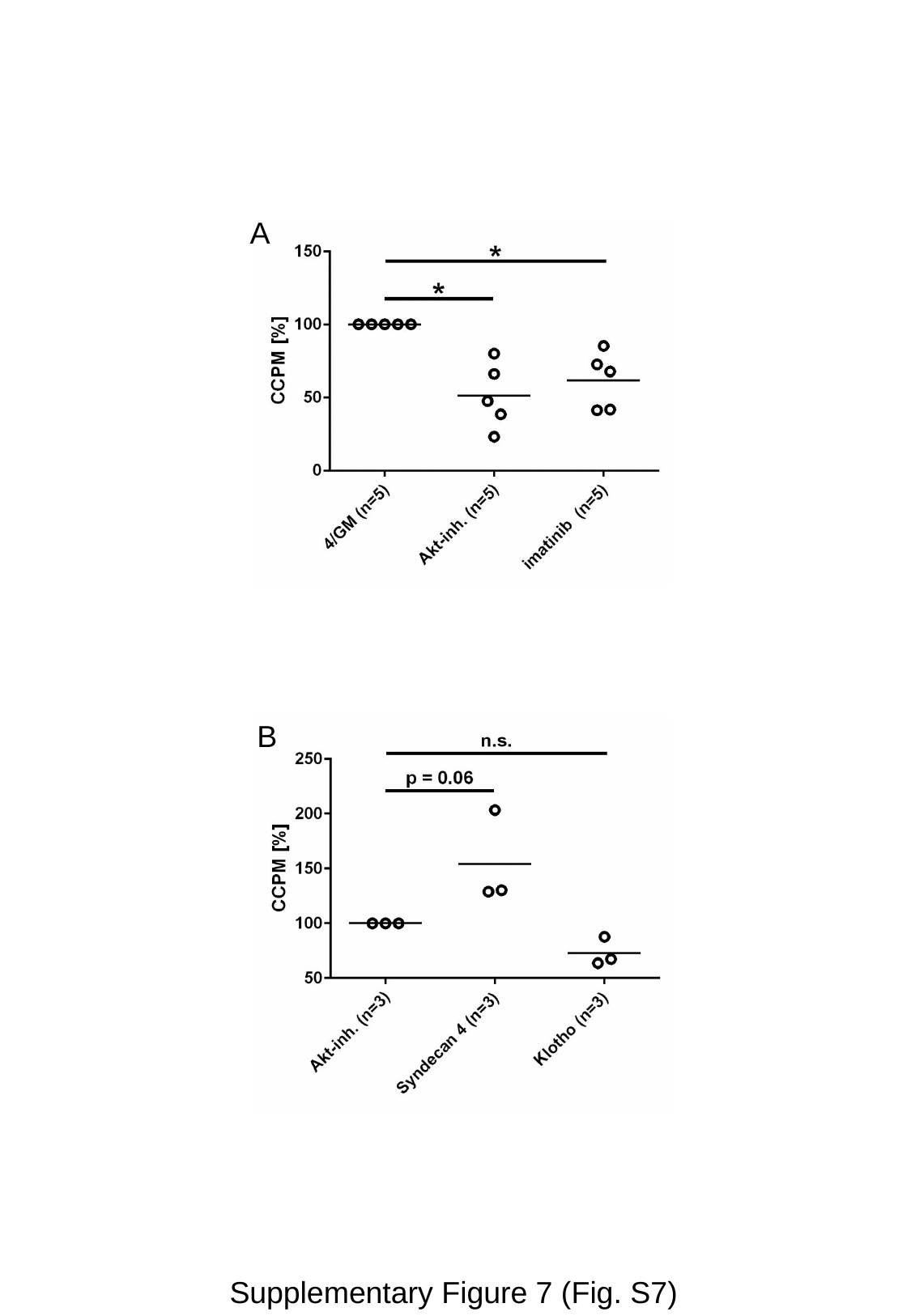

A
B
Supplementary Figure 7 (Fig. S7)

Supplement: Additional file 7: Figure S7. — Akt inhibition reduces the capacity of human moDC to induce T cell responses. Combined analysis of different donors. moDC generated in vitro with GM-CSF and IL-4 alone (4/GM) or with imatinib (3 μM) or Akt inhibitor MK2206 (Akt.-inh., 300 nM) were used as stimulators in MLR with allogeneic T cells. T cell proliferation was measured by [3H]thymidine incorporation. CCPM = corrected counts per minute. (A) Combined analysis of 5 different donors. The values were normalized to 100% for IL-4 and GM-CSF treated moDC. (B) Increasing concentration (0.0 μg/mL - 20.0 μg/mL) of blocking soluble recombinant T cell ligand SD-4 were added with recombinant Klotho β serving as control. Combined analyses of 3 different donors. The values were normalized to 100% for Akt inhibitor MK2206 (Akt.-inh., 300 nM) treated moDC. The raw data were used to perform Student’s t-test (ratio paired, two-sided, equal variance). Stars indicate significance (*P < .05, n.s. = not significant; absolute data were analyzed using Student’s t-test (ratio paired, one-sided, equal variance). [file 12964_2015_99_MOESM7_ESM.ppt]
