# Supplementary material for: The transcription factor MITF is a critical regulator of GPNMB expression in dendritic cells
Source: Cell Commun Signal. 2015 Mar 24;13:19. doi: 10.1186/s12964-015-0099-5 (PMC4422548; doi:10.1186/s12964-015-0099-5)
Supplement: Additional file 6: Figure S6. — MITF-Inhibition decreases GPNMB cell surface protein on moDC generated with imatinib or nilotinib. moDC were generated in vitro with GM-CSF, IL-4 and imatinib or nilotinib alone and additional MITF inhibitor ML329 (MITF-inh.; 2000 nM) or KLF5 expression inhibitor CID (2000 nM) as control. GPNMB protein level of CD209+ moDC was analyzed by flow cytometry. Data were analyzed using FlowJo software and histogram overlays are displayed as %Max, scaling each curve to mode = 100%. [file 12964_2015_99_MOESM6_ESM.ppt]

## Slide 1
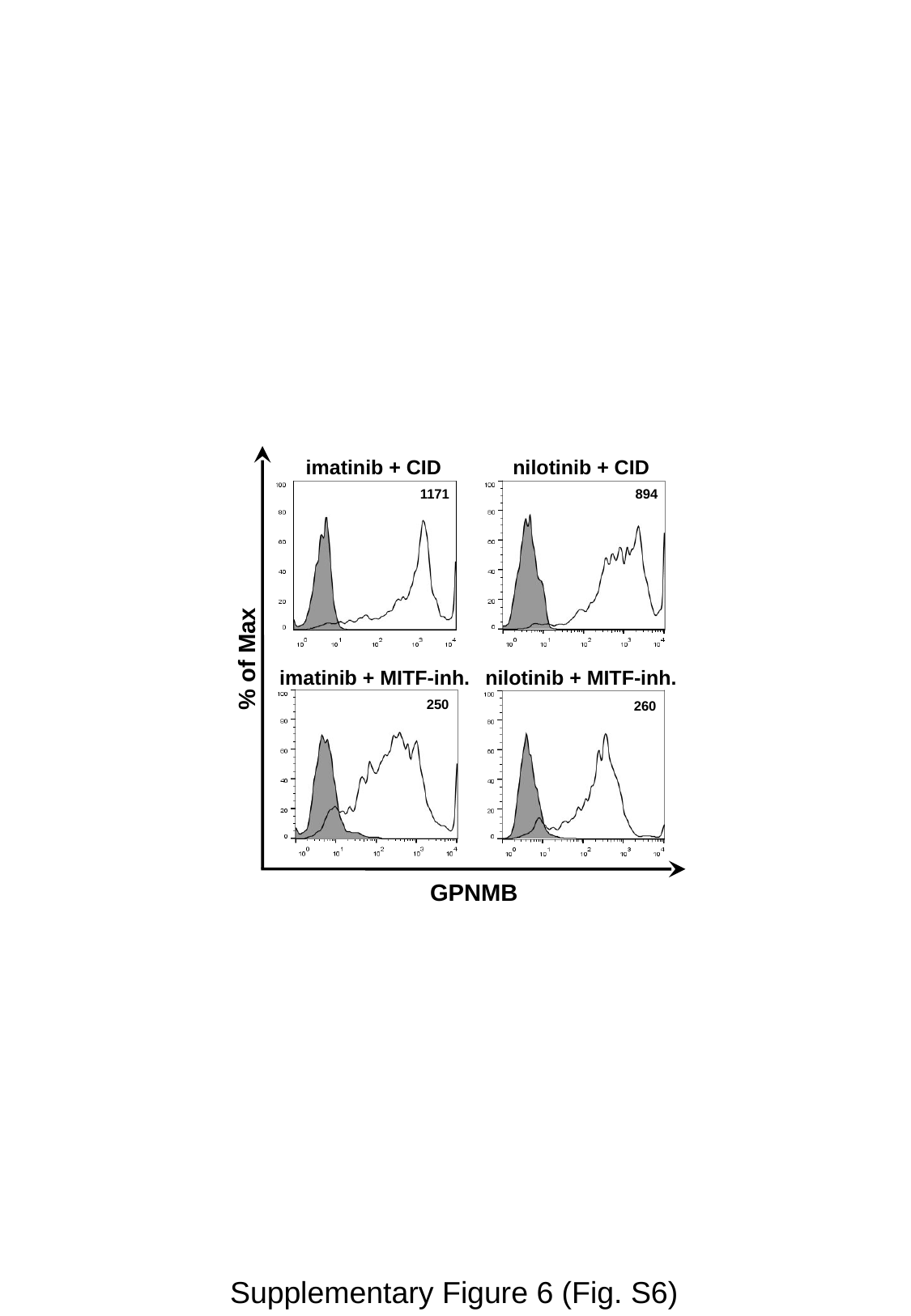

imatinib + CID
nilotinib + CID
1171
894
% of Max
imatinib + MITF-inh.
nilotinib + MITF-inh.
250
260
GPNMB
Supplementary Figure 6 (Fig. S6)
